# Relationship of Tijuana River Flow and Ocean Bacteria Counts and Emergency Department Diarrhea Cases

**DOI:** 10.5811/westjem.41492

**Published:** 2025-07-17

**Authors:** Jaya Jost, Conor Youngblood, Peter Jost, Roberto Medero

**Affiliations:** * Miramar College, San Diego, California; † University of California, Berkeley, Berkeley, California; ‡ Scripp Mercy Chula Vista, Department of Emergency Medicine, Chula Vista, California

## Abstract

**Introduction:**

The Tijuana River, which affects southern San Diego Beaches, is severely contaminated with untreated sewage. Exposure to pathogens can lead to various health problems, commonly gastrointestinal (GI) illnesses. We aimed to look for any relationship between Tijuana River flow rates and ocean pollution levels and levels of diarrhea at a nearby Emergency Department (ED).

**Methods:**

In this retrospective study that spanned the 2023 dry season and included Hurricane Hillary, we compared Tijuana River flow rates and fecal bacterial counts on the southern San Diego County coastline to the number of visits to a nearby ED, specifically a 225-patient sample size, with the chief complaint of diarrhea, a potential waterborne illness.

**Results:**

In late August of 2023, after Hurricane Hillary made landfall as a tropical storm in Baja California, Mexico, there was a large increase in the Tijuana River flow rate and a correspondingly significant increase in diarrhea cases at 3.25 times the mean, from a mean of 4.25 cases per week to 14 cases the week of Hurricane Hillary.

**Conclusion:**

We found a significant correlation between Tijuana River transboundary flow rates and Emergency Department case levels of diarrhea, a known waterborne illness, in the summer of 2023.

## INTRODUCTION

Imperial Beach, a coastal community in southern San Diego County, CA, is five miles north of the Mexican border. Imperial Beach has had its coastal waters closed to the public for more than 1,000 consecutive days since 2022 related to high levels of cross-border sewage contamination. Inadequate sewage treatment facilities with aging infrastructure to support the growing population in the city of Tijuana, Mexico, have allowed sewage, agricultural, and urban runoff to pollute the Tijuana River Valley, These contaminants ultimately discharge into the Pacific Ocean and are carried toward Imperial Beach and as far north as Coronado, nine miles from Imperial Beach. The San Diego Department of Environmental Health and Quality tests the ocean water off Imperial Beach daily and publishes the results on www.waterboards.ca.gov. The water is tested for fecal indicator bacteria (FIB) such as *Escherichia coli* and *Enterococcus*.

The Tijuana River is contaminated with sewage, industrial waste, and urban runoff. It is has been identified as an impaired water body, per the US Clean Water Act, and a public health threat with health implications.^1^,^2^,^3^,^4^,^5^,^6^ The presence of FIB and, correspondingly, its concentration is indicative of more virulent bacteria and viruses being present in both the seawater and sea spray aerosol.^7^,^8^,^9^ Exposure to these pathogens can lead to various health problems, commonly gastrointestinal illness including enterovirus and norovirus, as well as respiratory, eye, ear and skin problems, that prompt medical visits.^10^,^11^,^12^ It is estimated that 7.15 million waterborne illnesses occur annually in the United States.^13^ Even sand from contaminated beaches may be a route of exposure.^14^ In this study we aimed to investigate Tijuana River flow rates and FIB levels in the ocean off southern San Diego County and any correlation with the frequency of potential waterborne-related healthcare visits to the emergency department (ED), specifically for diarrheal illnesses.

## METHODS

We looked for a correlation between Tijuana River flow rates and FIB levels on the Imperial Beach coastline by obtaining the river flow rates and FIB levels from waterdata.ibwc.gov and waterboards.ca.gov. We also obtained visit data on the number of patients seen for a chief complaint of diarrhea in US ZIP codes 92118, 91932, and 92154 (Coronado, San Ysidro, and Imperial Beach) using Epic Slicer Dicer, a data analysis tool in the electronic health record system (Epic Systems Corporation, Verona, WI). We looked for a temporal correlation between visit numbers and Tijuana River flow-rate volume and FIB levels at Imperial Beach in 2023. This was a retrospective population study, and all data analyzed is free of any patient identifiers.

## RESULTS

In 2023 we correlated residential ZIP codes 92118, 91932, and 92154 with patient healthcare visits to an ED in the neighboring city of Chula Vista. We found 225 patients who were seen for chief complaints of diarrhea over 52 weeks. The mean was 4.3 patients per week. There was a spike in diarrhea cases seen from August 20–26, 2023 to 14 cases, 3.25 times the mean. This was the week that Hurricane Hilary made landfall as a tropical storm in Baja California bringing torrential rain and causing much higher volumes of Tijuana River flow at 354.8 million gallons average daily transboundary flow. (The yearly weekly average for 2023 was 118 million gallons; summer season 36.5 million gallons).

Data is limited with regard to FIB levels as the county does not tend to test on days that they know the counts will be higher based on rainfall levels. For example, two days after Tropical Storm Hilary, Silver Strand State Beach total coliforms reached >16,000 copies/100 milliliters (mL). Imperial Beach did not have any levels reported that week, but the beach remained closed. Coronado Beach 8.9 miles to the north went from 916 copies/100mL to 32,094 copies from August 16 to August 24. The highest flow rate days between were not tested.

We focused our analysis on the summer months when ocean activity is higher and the risk of exposure to waterborne illnesses increases. Specifically, we examined the drier period from June 4–November 18, 2023. By correlating the daily average transboundary flow of the Tijuana River with patient encounters reporting diarrhea as the chief complaint, we identified a strong correlation, with a Pearson coefficient of .75. (See Figures 1A and 1B).

**Population Health Research Capsule**

What do we already know about this issue?

*The Tijuana River and nearby ocean is contaminated with sewage; exposure to sewage pathogens can lead to health problems, commonly gastrointestinal illnesses*.

What was the research question?

*Is there a correlation between Tijuana River flow/ocean pollution levels and cases of diarrhea in the local population?*

What was the major finding of the study?

*We found a Pearson correlation coefficient of .75 between transboundary Tijuana River flow and diarrhea cases at a local ED in the summer of 2023*.

How does this improve population health?

*Increased awareness of the risks of polluted ocean water would help protect residents in coastal southern San Diego County communities*.

## DISCUSSION

Looking at both FIB and Tijuana River flow rates, the data on FIB levels provided on waterboard.ca.gov is incomplete. Although we did not evaluate for a definitive correlation between transboundary flow rate and FIB levels, there is an obvious trend when looking at the data. The Tijuana River flow rate is more useful as a daily indicator of possible water contamination levels, as it is reported every day. Looking at summer dates in 2023, we noted a spike in the number of ED visits with a chief complaint of diarrhea in patients with residential South Bay ZIP codes the week of August 20, 2023, which corresponded with a dramatic spike in the transboundary river flow. The Pearson correlation coefficient of .75 is strong. Diarrhea cases increased by 325% from mean summer levels. We hope increased awareness of the dangers of polluted ocean water will help protect residents in the coastal southern San Diego County communities.

## LIMITATIONS

Limitations of this study include the fact that many people who get ill from the water do not seek medical attention. Another limitation is that winter dates with high flow rate and more frequent rains do not correlate with levels of waterborne illness and diarrhea, as most people do not go into the water in colder months. We found no correlation between flow rates, FIB levels and diarrhea in winter months, or when looking at 2021 and 2022. Our finding may be a unique event in August 2023 coinciding with a tropical storm. Another limitation is we do not know whether the patients had ocean exposure.

Future studies could include a prospective survey following an ocean-user population, to track levels of illness and compare against transboundary flow rates and/or FIB. Again, we found FIB levels to be a less reliable indicator, as it is not updated or checked every day. It tends to not be measured on days when the levels are predicted to be high, possibly to ensure the safety of testers. However, on those days we did find that the beaches were closed without levels checked. A final limitation to this study is that the patient data abstractor was not blinded to our hypothesis.

## CONCLUSION

We found a correlation between Tijuana River transboundary flow rates and an increased number of visits to the ED with a chief complaint of diarrhea, a known waterborne illness, in southern San Diego County in the summer of 2023, with a peak number of cases reported the week that Tropical Storm Hilary made landfall in the Mexican state of Baja California.

## Figures and Tables

**Figure 1A:**
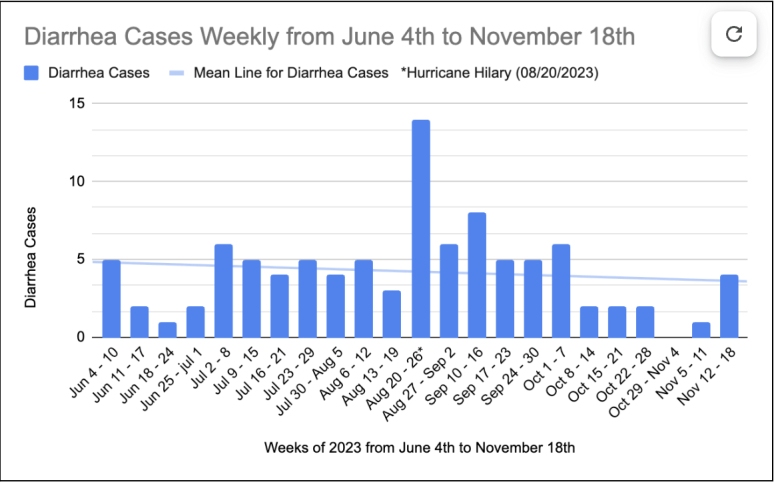
Emergency department cases for chief complaint of diarrhea from June 4–November 18, 2023.

**Figure 1B:**
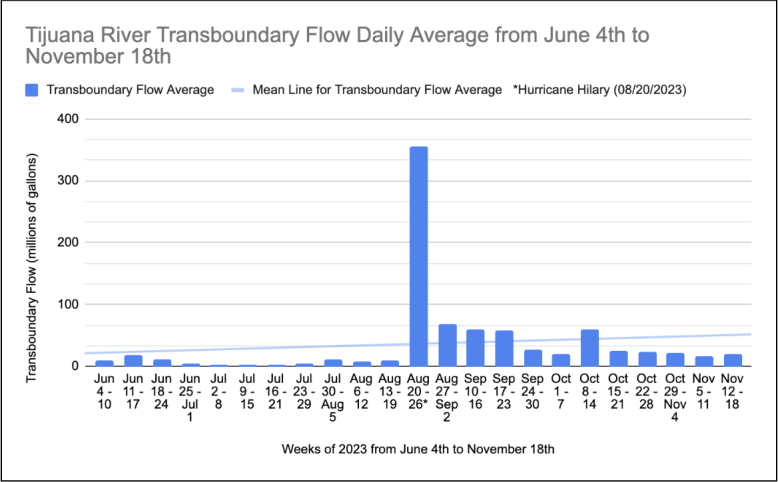
Tijuana River flow average at US-Mexico border in millions of gallons of water per day from June 4–November 18, 2023.
